# Biotechnological Aspects and Perspective of Microbial Keratinase Production

**DOI:** 10.1155/2015/140726

**Published:** 2015-06-09

**Authors:** Subash C. B. Gopinath, Periasamy Anbu, Thangavel Lakshmipriya, Thean-Hock Tang, Yeng Chen, Uda Hashim, A. Rahim Ruslinda, M. K. Md. Arshad

**Affiliations:** ^1^Institute of Nano Electronic Engineering (INEE), Universiti Malaysia Perlis, 01000 Kangar, Perlis, Malaysia; ^2^Advanced Medical & Dental Institute (AMDI), Universiti Sains Malaysia, 13200 Kepala Batas, Penang, Malaysia; ^3^Department of Oral Biology & Biomedical Sciences and OCRCC, Faculty of Dentistry, University of Malaya, 50603 Kuala Lumpur, Malaysia; ^4^Department of Biological Engineering, College of Engineering, Inha University, Incheon 402-751, Republic of Korea

## Abstract

Keratinases are proteolytic enzymes predominantly active when keratin substrates are available that attack disulfide bridges in the keratin to convert them from complex to simplified forms. Keratinases are essential in preparation of animal nutrients, protein supplements, leather manufacture, textile processing, detergent formulation, feather meal processing for feed and fertilizer, the pharmaceutical and biomedical industries, and waste management. Accordingly, it is necessary to develop a method for continuous production of keratinase from reliable sources that can be easily managed. Microbial keratinase is less expensive than conventionally produced keratinase and can be obtained from fungi, bacteria, and actinomycetes. In this overview, the expansion of information about microbial keratinases and important considerations in keratinase production are discussed.

## 1. Introduction

**Figure 1:**
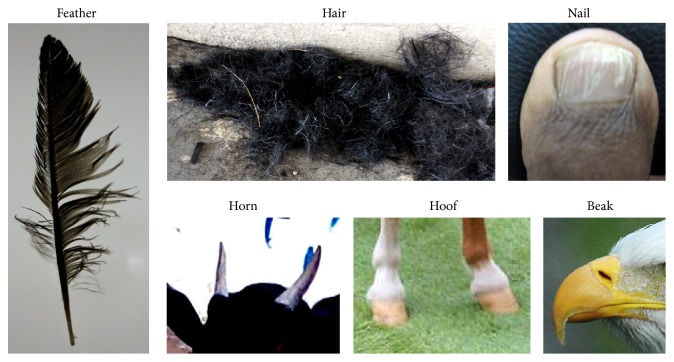
Sources of keratin. Different sources such as feathers, hair, nails, horns, hooves, and beak are shown. The hosts for these sources include human, bird, and animal. The hardness of these keratin materials is different in each case.

**Figure 2:**
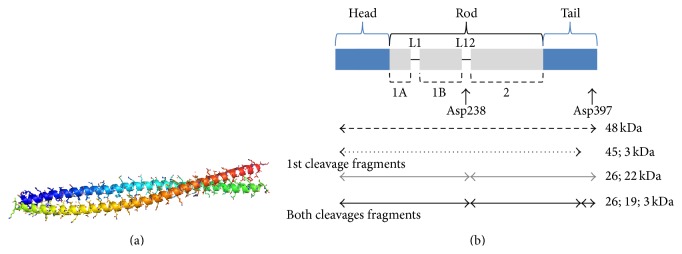
(a) Crystal structure of K5 and K14 coil heterocomplex (PDB accession code: 3TNU). This is a heteromeric assembly and perinuclear organization of keratin filaments. Regions from central-coiled domains of two filaments are interacting. (b) Representation of K18 caspase-cleavage sites during apoptosis. Keratin network regulates apoptotic machinery and confers a caspase-activation. The primary caspase-targets in epithelial cells are found in keratins type I family (reproduced from [2]).

Keratin is one of the most abundant biopolymers in the world [1]; it is a tough, fibrous, insoluble material that functions as an outer coat of human and animal organs, to prevent the loss of body fluids. Keratin is predominantly found in tissues of reptiles, birds, amphibians, and mammals. The structural component of feathers, hair, nails, horns, hooves, bones, furs, claws, hides, bird beaks, skin, wool, scales, and bristle is made up of keratin (Figure 1). *α*-keratins (alpha-helix) are usually found in the hair, wool, horns, nails, claws, and hooves of mammals, whereas the harder *β*-keratin (beta-sheets) is found in bird feathers, beaks, and claws. Keratin is also expressed in the epithelial cell types of digestive organs (liver, pancreas, intestine, and gallbladder), which include hepatocytes, hepatobiliary ductal cells, oval cells, acinar cells, enterocytes of the small intestine, colon, and goblet cells [2]. Keratin is rich in sulfur compounds with disulfide bridges, which imparts them with an insoluble nature. It also contains a variety of amino acids, predominantly cystine, lysine, proline, and serine. Keratin is hard, containing scleroprotein, while it is unreactive against most chemicals and is not digested by pepsin, trypsin, or papain [3]. Higher vertebrates, including humans, cannot digest keratinous materials. Keratin is a monomer that forms bundles of intermediate filaments that are expressed in epithelial cells that have been linked to human liver diseases. Structural details regarding keratin filaments 5 and 14 for heteromeric assembly and perinuclear organization have been reported ([4], Protein Data Bank Accession code: 3TNU; Figure 2(a)). Among different keratin filaments, K8 and K18 are important for the protection of hepatocytes [2]. The representation of K18 caspase-cleavage sites during apoptosis has been described in detail ([2], Figure 2(b)).

The major sources of keratin accumulation, which cause environmental problems, initiate from industries that use keratin as the raw material. Poultry farms are also involved in dumping of feather wastes (barbs and rachis). Indeed, 90% of feathers are keratin, and millions of kilograms of feathers are discarded to the environment annually [6]. The disposal of feathers is also accompanied by natural falling of feathers and hairs from birds during production, so it is necessary to develop methods to reduce keratin accumulation. For environmental remediation of keratin, an immediate step that has easy processing set-up with lower cost is desired. Microbial keratinase may meet these preferences, as keratinophilic fungi, bacteria, and actinomycetes naturally reside on keratin wastes. Here, we elaborated the currently available information pertaining to microbial keratinase production.

## 2. Keratinophilic Fungi

Keratinophilic fungi produce the proteolytic enzymes that are capable of decomposing keratinic waste materials [7]. Several keratinophilic fungi that live as parasites on keratinous materials use keratin as their carbon and nitrogen sources, multiply in an asexual manner, and produce conidia. During the process of fungal colonization, boring hyphae are produced to drill into the keratin substrate. These keratinophilic fungi include hyphomycetes and several other taxa [8]; hyphomycetes include both dermatophytic (e.g.,* Microsporum* species) and nondermatophytic (e.g.,* Chrysosporium* species and other genera) keratinophilic fungi [9]. The dermatophytes are mainly from the genera* Microsporum, Epidermophyton,* and* Trichophyton*. Keratinophilic species are usually identified by morphological features of their macro- and microconidia, molecular methods, and using DNA sequence analysis [10]. Keratinophilic fungi produce sulfide for sulphitolysis and, during this process, the disulfide bonds of cysteine, a major amino acid in keratinous materials, are broken down, after which the proteolytic enzymes released by the fungi can easily cleave the keratin. During the degradation process, the products released are cysteine, S-sulphocysteine, cysteine acid, cysteine, and inorganic sulfate, and the presence of these products in the culture media indicates the occurrence of true keratinophilic fungi. Fungi that do not show this behavior during degradation are considered nonkeratinophilic fungi. Keratinophilic fungi are predominantly anthropophilic (human loving) or zoophilic (animal loving). Many keratinophilic fungi have been isolated from soil samples due to accumulation of keratin wastes in the soils (geophilic). Soil samples from geophilic habitats including public beaches, agricultural areas, public parks, gardens, and elementary schools have been found to contain keratinophilic fungi [11–15]. Most of these studies involved an isolation technique known as keratin-baiting, in which hair or feathers are used for the isolation of keratinophilic fungi [11–15]. Keratinophilic fungi isolated in countries worldwide, including Egypt, Spain, Australia, Palestine, Kuwait, India, Iran, and Malaysia, have been described [8].

The common isolates of keratinophilic fungi from soils include* Microsporum gypseum, M. canis, M. fulvum, M. nanum, Trichophyton terrestre, T. ajelloi, T. mentagrophytes, T. interdigitale, T. verrucosum, T. equinum, T. rubrum, T. interdigitale, T. schoenleinii, T. simii, Chrysosporium keratinophilum, C. pannicola, C. tropicum, C. indicum, C. anum, C. lobatum, C. evolceanui,* and* C. indicum*. Shadzi et al. [12] have collected 330 samples from thirteen elementary schools and seven public parks and identified 214 species, among which* Chrysosporium keratinophilum* was the dominant organism, being present with a frequency of 54.2%. Anbu et al. collected 10 and 12 soil samples from poultry farms and feather dumping locations, respectively, and recovered 34 fungal species belonging to 19 genera. Among these, six species are dermatophytes belonging to five genera [13]. Kachuei et al. [15] analyzed 800 soil samples from Isfahan province of Iran and found that 588 belong to keratinophilic fungi, representing 73.5% of the total isolates. Furthermore, they recovered 16 species belonging to 11 genera. Similarly, 108 soil samples from St. Kitts and 55 samples from Nevis were shown to consist of 49 and 38 samples, respectively, positive for keratinophilic fungi. Additionally,* M. gypseum* was predominantly found in 15.7 and 40% of soils of these collections sites, respectively, followed by* Chrysosporium* species [14]. Molecular identification of keratinophilic fungi revealed 411 isolates from 22 genera in public park soils from Shiraz, Iran [9]. Another study revealed that 48 soils from Jharkhand, India, contained 10 species of keratinophilic fungi belonging to seven genera [8]. Similarly, 500 samples collected from zoos and parks of Ahvaz were found to contain keratinophilic fungi [16]. In another study, 54 soil samples from different collection sites including gardens, schools, poultry farms, rivers, hospitals, and garbage dumping sites were found to contain 23 species of keratinophilic fungi from 11 genera. The abundance of samples shown to contain keratinophilic fungi was as follows: 65% gardens, 52% schools, 43% poultry farms, 34% garbage, 30% hospitals, and 21% rivers [7]. Based on the above studies, it is clear that keratinophilic fungi are ubiquitous and present in all kinds of soils and that they are dominant in areas where humans and animals live. In addition to the above list, keratinolytic proteins from keratinophilic fungi were reported by Yu et al. [17], Asahi et al. [18], and Williams et al. [19].

## 3. Keratin-Degrading Bacterial Isolates

Similar to the isolates of fungi, lists of bacterial strains capable of degrading keratins have been reported. Bacteria can grow faster than fungal species and therefore have potential in industrial applications. The advantages of fungi include easier colonization of fungal hyphae into the harder keratin relative to bacteria. The isolated bacterial strains known to degrade keratin or produce the keratinase are primarily composed of* Bacillus*; it includes* B. subtilis *and* B. licheniformis* [20], although other bacteria including Gram-positive* Lysobacter, Nesterenkonia, Kocuria,* and* Microbacterium* and Gram-negative* Vibrio, Xanthomonas, Stenotrophomonas*,* Chryseobacterium, Fervidobacterium, Thermoanaerobacter,* and* Nesterenkonia* can also degrade keratin ([21] and references therein). Several other studies have investigated keratinase produced by bacterial species [22–26]. Sapna and Yamini [27] investigated the potential degradation of keratin by bacterial strains recovered from the soil samples. Four isolates from feather waste were recovered on milk agar plates and three were identified as Gram-negative bacteria (*Burkholderia, Chryseobacterium,* and* Pseudomonas* species) and one was identified as Gram-positive strain (*Microbacterium* species) [28]. Moreover, Korniłłowicz-Kowalska and Bohacz [29] reported that actinomycetes,* Streptomyces* group, namely,* S. fradiae, Streptomyces* species A11,* S. pactum, S. albidoflavus, S. thermoviolaceus* SD8, and* S. graminofaciens*, as well as* Thermoactinomyces candidus*, were capable of producing keratinase.

## 4. Secretion of Microbial Keratinases

**Figure 3:**
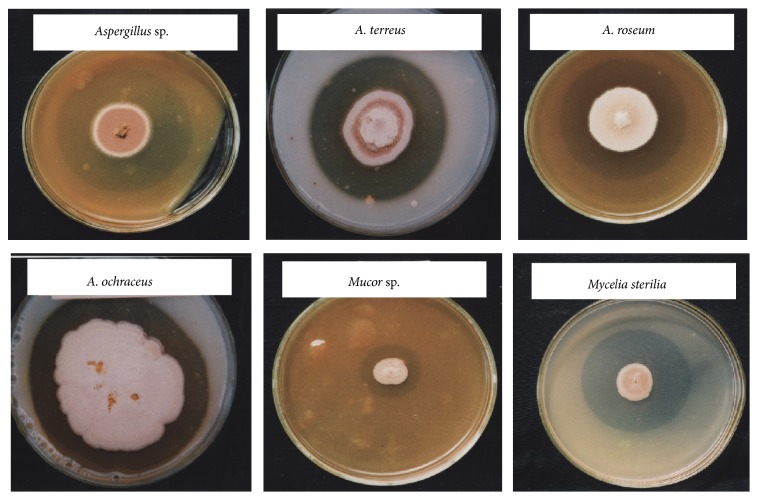
Plate clearance assay for proteolytic activity. Secretion of proteolytic enzymes by* Aspergillus* species,* Mucor* species, and* Mycelia sterilia* is shown as example. The 8% gelatin agar plates were prepared and a pinpoint inoculum was spotted at the center. The clear zone around the colonies indicated the presence of proteolytic activity, which was due to the complete degradation of gelatin. Aqueous saturated solution of ammonium sulfate was added on the surface of the agar for clear visualization.

Keratinolytic enzymes are proteases known as keratinases (EC 3.4.21/24/99.11) that can primarily be obtained from fungi, actinomycetes, and bacteria [29]. Fungal keratinases can be easily obtained by secretion, and their low cost makes them preferable over bacterial keratinases in some cases, even though the fungi grow slower and the recovery of keratinase from fungi has been reported for several decades. The availability of several strains that are capable of producing keratinase makes the situation to select efficient keratinase producers an important step. Screening microbial enzymes is essential in the selection process, and the chosen enzymes should be less expensive, eco-friendly, and efficient. Both keratinophilic fungi and nonkeratinophilic fungi can produce keratinases, but the difference is the rate of production, which is higher in the former case. Several methods have been proposed to screen proteolytic (including keratinolytic) enzymes, including keratin-baiting, plate screening, spectrophotometric methods, and sequence-based amplification. Jeevana Lakshmi et al. [30] identified feather-degrading bacteria using the 16S rDNA sequence. Among the aforementioned methods, the plate-clearing assay is one of the popular methods due to displaying visual results, as well as being less expensive and easier than other methods (Figure 3). The keratin-baiting method is used for the initial screening and isolation of keratinolytic species. In this method, any keratin source can be the bait; hair and feathers are routinely in use [11, 13]. Even though the pour plate method can be used to isolate the keratinophilic microbes as an alternate, keratin-baiting is also commonly applied because it enables the direct selection of keratinophilic species on the substrate.

## 5. Optimized Conditions for Microbial Keratinases

Once microbes are isolated, they can be further cultivated on suitable artificial growth media under optimal conditions to obtain excess production of keratinase. Sabouraud's dextrose is commonly used to grow keratinophilic fungi due to its suitability [11, 13, 16]. Usually keratinophilic fungi will take a longer time to degrade the keratin (in weeks). Using the hair-baiting technique, Gugnani et al. [14] found that 4 to 8 weeks were required to observe keratinophilic fungal growth. Kumar et al. [8] isolated keratinophilic fungi after 2 to 4 weeks of incubation, while Mahmoudabadi and Zarrin [16] found that 4 to 5 weeks are necessary to grow. In such cases, optimal growth was found to occur at room temperature. It has also been reported that keratinophilic fungi are able to degrade 40% of keratin after 8 weeks, while less than half (<20%) of that amount can be degraded in the case of nonkeratinophilic fungi [31].

It has been reported that most keratinophilic microbes thrive well under neutral and alkaline pH, the range being 6.0 to 9.0 [32]. Most keratinophilic fungi are mesophiles, although* M. gypseum* and some species of* Chrysosporium* are thermotolerant ([29] and references therein). It has been reported that temperatures of 28°C to 50°C favor keratinase production by most bacteria, actinomycetes, and fungi, while 70°C favors its production by* Thermoanaerobacter* and* Fervidobacterium* species [33–35]. Optimal keratinase production by* Chrysosporium keratinophilum* occurs at 90°C and its half-life is 30 min [36], whereas the thermophile* Fervidobacterium islandicum* AW-1 has an optimum of 100°C and a half-life of 90 min [35].

The complete optimized conditions for microbial keratinases production are described in detail elsewhere [37]. Under optimal condition, keratinophilic fungi,* Scopulariopsis brevicaulis* and* Trichophyton mentagrophytes*, result in keratinase activity to the levels 3.2 and 2.7 Keratinase Unit (KU)/mL with the ability to degrade 79 and 72.2% of chicken feathers, respectively [38]. Matikeviciene et al. [20] have shown keratinase activity of 152 KU/mL after 24 h of incubation using* Bacillus* species with optimal media. Higher amounts of keratinase were reported by Kanchana [3] at 37°C for 72 h in medium containing feather meal and 0.025% yeast extract at a pH 7.0 under submerged culture. Laba and Rodziewicz [39] optimized the conditions for keratinolytic feather-degrading ability of* Bacillus polymyxa* and* B. cereus*. Additionally, Sivakumar et al. [40] recently optimized the culture conditions for the production of keratinase from* Bacillus cereus* TS1. Using dimeric keratinase obtained from* Bacillus licheniformis* ER-15 complete degradation was achieved within 8 h at pH 8 and 50°C. In this case, 25 g of chicken feathers was degraded with 1200 KU [41].

## 6. Purification of Keratinases

**Figure 4:**
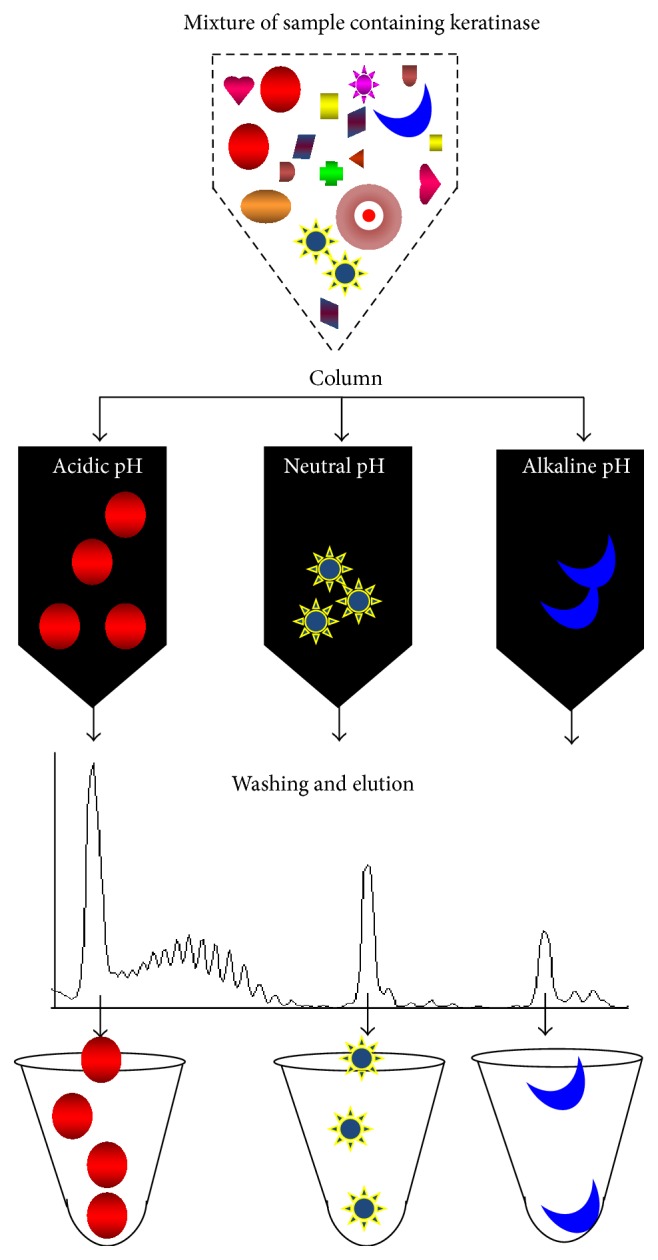
Purification strategy for keratinases. Options with acidic, neutral, and alkaline keratinases are shown. Peak profiles indicate the individual proteins. Conventional purification strategies include ammonium sulphate precipitation followed by ion-exchange and gel-filtration. Other methods such as immunochromatography, high-performance liquid chromatography, and fast protein liquid chromatography are also involved in the purification of keratinases.

In addition to the higher keratinase production under optimal conditions, purification of keratinase is necessary for further industrial applications to hasten the efficiency of keratinase action. Molyneux [42] attempted to isolate keratinase from a bacterial source. In other cases, with the purified keratinases, several sizes were reported in the apparent molecular weight range of 27 to 200 kDa from different strains of bacteria and fungi ([29] and references therein). However, Kim et al. [43] reported recovery of keratinase with a molecular weight of 440 kDa. Purified enzymes including keratinases can be obtained using different methodologies. The most common strategy is to purify the enzymes by precipitation followed by column chromatography. Keratinase with a molecular mass of 35 kDa was purified from feather-degrading bacterium using ammonium sulphate precipitation followed by ion-exchange (DEAE-Sepharose) and gel-filtration (Sephadex G-75). The purified keratinase was found to have thermotolerant and showed high specific activity [44]. Using a similar strategy, Zhang et al. [45] purified the alkaline keratinase from* Bacillus* species and identified keratinase of 27 kDa using MALDI-TOF-MS. Anbu et al. [5] isolated keratinase with a molecular weight of 39 kDa from the poultry farm isolate,* Scopulariopsis brevicaulis*, and found that this keratinase had a serine residue near the active site. Keratinase with a size of 41 ± 1 kDa and activity under the optimal conditions at pH 9.0 and 50°C was isolated from* Bacillus megaterium*. This enzyme was also found to have a serine active site and to be inhibited by PMSF [46]. Based on the pH adaptation nature of the keratinase, the column matrix and method of purification can be desired while varying the elution profile (Figure 4). In addition, keratinase purification can also be accomplished with greater efficiency by immunoprecipitation when the appropriate anti-keratinase antibody is available. Similarly, immunochromatography technique can be implemented using anti-keratinase antibody for the efficient purification of keratinase. Purified keratinases from diverse species have displayed higher stability under varied condition (Table 1).

## 7. Acceleration of Microbial Keratinase Production

**Figure 5:**
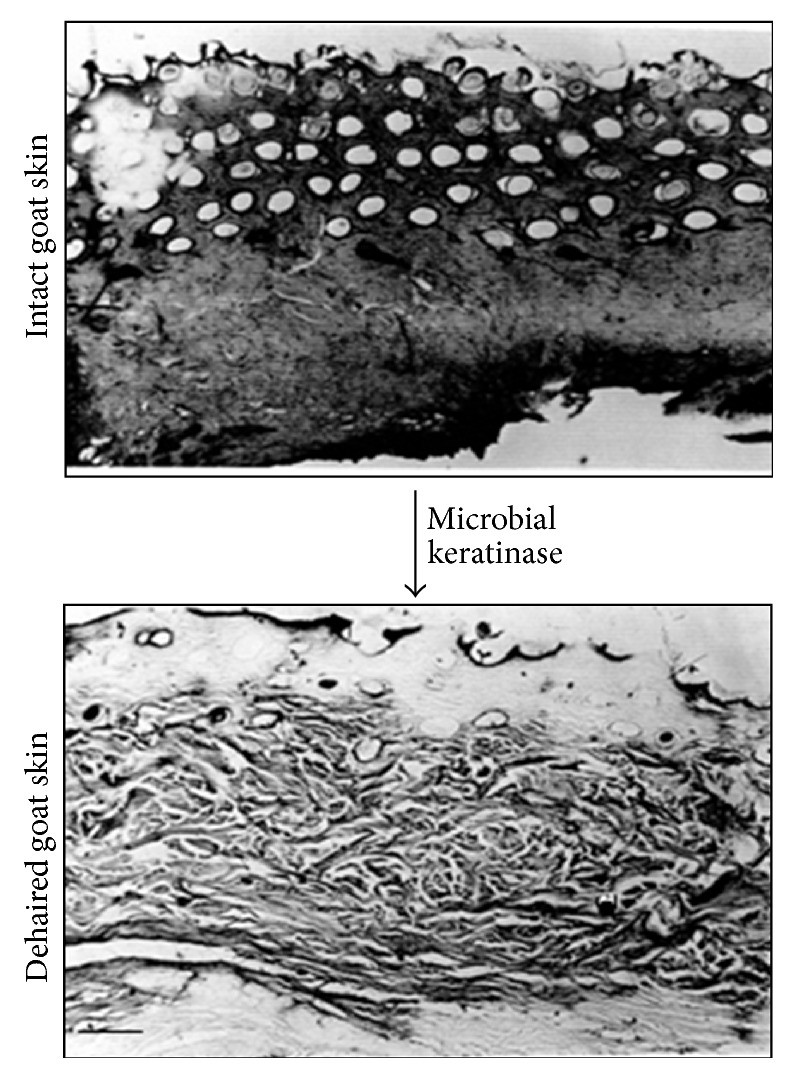
Dehairing using microbial keratinase (2 KU/mL) produced by* Scopulariopsis brevicaulis* (source from [5]). The purified keratinase was sprayed on the flesh side of the skin and then folded and incubated for 30 days. Every 3 days of interval, the dehairing ability was examined.

Following the optimization of the basic conditions for keratinase production and purification, it is necessary to accelerate overproduction of keratinase. This can be accomplished by recombinant DNA technology and statistical optimization. Sequences for both the substrate-keratin and the enzyme-keratinase have been proposed. The primary sequences of the keratin involved in its recombinant production were found by Hanukoglu and Fuchs [60, 61] and denoted by type I and type II. Later, several amino acids sequences for keratinase were revealed. The amino acid sequence of keratinase from* Bacillus licheniformis* and other species is available in data bank ([62], e.g., accession code AAB34259). Similarly, the full length of keratin sequences from* Homo sapiens* has been reported ([63], accession code P04264). For the large-scale preparation of keratinase, recombinant DNA technology would yield a large amount of overexpressed enzyme. Recombinant or other keratinases purified using conventional methods have great potential for applications in industrial processes such as dehairing. For example, Anbu et al. [5] have accomplished dehairing using purified keratinase from the keratinophilic fungi,* Scopulariopsis brevicaulis* (Figure 5).

**Figure 6:**
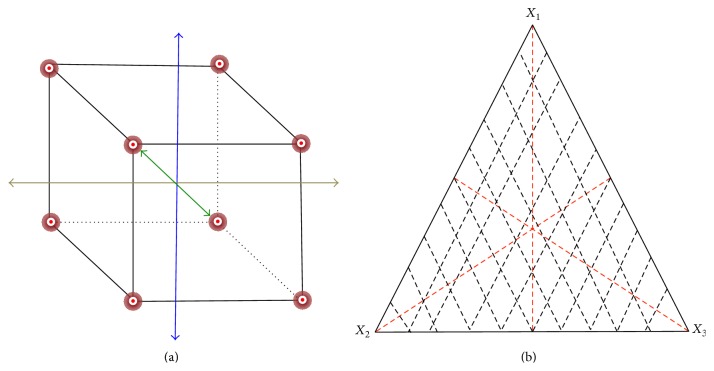
Basic strategy for statistical optimization of keratinase. (a) Response surface methodology perturbation plot; (b) mixture trace plot. Response surface methodology is a collection of statistical techniques for designing experiments, building models, and evaluating the effective factors. It is an efficient statistical technique for optimization of multiple variables to predict best performance conditions with minimum number of experiments.

The production levels of any given enzyme can also be improved severalfold using statistical modeling studies. There are different formulations of statistical calculations with basic formulae that have been described. Some basic models for optimization are given in Figure 6, which shows a response surface methodology perturbation plot and mixture trace plot. One of the basic models, the Box-Behnken design, is related to experimental variables by the response equation:

(1) $$\begin{array}{c} Y=f\left(X_{1},X_{2},X_{3},\ldots ,X_{k}\right). \end{array}$$

A second-degree quadratic polynomial is then used to represent the function by

(2) Y=R0+∑i=1kRiXi+∑i=1kRiiXi2+∑i=1,i<jk−1 ∑j=2kRijXiXj+ε.

The variables and other parameters have been described previously in detail [64]. Using a statistical optimization model, Harde et al. [65] optimized the keratinase production of* Bacillus subtilis* NCIM 2724. These authors used one-factor-at-a-time optimization and an orthogonal array design. Recently, Shankar et al. [66] used response surface methodology, for the optimization of keratinase production by* Bacillus thuringiensis*. Using this design experiment, they compared the actual experimental and predicted calculated values and found that pH 10 and 50°C with 1% mannitol were ideal for keratinase production from* B. thuringiensis*. Similarly, Ramnani and Gupta [67] optimized the medium composition for the production of keratinase from* B. licheniformis* RG1 using response surface methodology. In another study,* B. cereus* was used for the study to optimize keratinase production [68]. Using the Box-Behnken design experiments, Anbu et al. [5] optimized the activity of purified keratinase from* Scopulariopsis brevicaulis* and achieved 100% activity with 5 mM CaCl_2_ at pH 8.0 and 40°C. Similarly, production of keratinase by* Scopulariopsis brevicaulis* and* Trichophyton mentagrophytes* has also been optimized using Box-Behnhen design experiments by Anbu et al. [38, 69].

## 8. Sensing Keratinases

In the above sections, various aspects regarding the conditions necessary for keratinase to degrade keratin are provided. However, detection strategies are also important for future applications of keratinase. Detection of keratinase or other biomolecules and their interactive analyses with binding partners can be accomplished using biosensors. Biosensors consist of a physicochemical detector and a biological component, enabling binding events to be transduced, thereby allowing detection of very small amounts of target biomolecules (keratinase). Sensors are broadly classified as electrochemical, electrical, optical and mass-sensitive, chemiluminescence, fluorescence, quantum dot-based, colorimetric, and mass spectroscopic detections. Different sensing surfaces can be adopted for the detection of keratinases. Developing sensing strategies for the detection of keratinase favors the analysis of keratinase from mixtures of a given sample.

Generally, gold- or silica-based sensing surfaces have been used to analyse the biomolecules [70–77]. To capture keratinase on these surfaces, appropriate tags can chemically modify keratinase. Thiol-modification of keratinase can enable its attachment onto the surface of gold or modification of the sensing surface with the COOH-terminal for ultimate attachment to amines on keratinase. Similarly, in the case of silica, surfaces must be chemically modified using amino-coupling agent followed by suitable tags, which can couple an amino group on the keratinase. In short, both gold and silica can be modified to capture keratinase, or keratinase can be modified for specific sensing surfaces as reported in other cases [70, 77]. Diverse keratinases from different species have been reported (Table 1) and these keratinases could be active at different pH and stable, indicating the suitability for various sensing systems.

## 9. Perspectives

Keratin, which is one of the most abundant hard materials in soil, is difficult to degrade under natural conditions. However, microbial degradation is an easier and less expensive method for conversion of these products to useful end products. Several methods to improve keratinase production have been suggested, and keratinase has been overexpressed, successfully purified, and applied to several industrial applications. In addition, additional developments have been implemented in keratinase research recently [78, 79]. However, there is currently no highly sensitive system available for the detection of keratinases. In addition, use of recombinant keratinase chimeras has the potential to generate efficient keratinase and needs to be improved. Development of more efficient methods for the production and detection of keratinase will hasten its application to industries and environmental waste management.

## Figures and Tables

**Table 1:** Keratinases from different species for various applications.

Species	Optimal condition (pH)	Aim(s) of study	Reference
Fungi			
*Aspergillus oryzae *	8.0	Purification and characterization	[47]
*Doratomyces microsporum *	8.0–9.0	Comparative analysis	[48]
*Paecilomyces marquandii *	8.0	Comparative analysis	[48]
*Trichophyton rubrum *	8.0	Purification and characterization	[18]
*Microsporum gypseum *	8.0	Secretion of keratinase	[49]
*Scopulariopsis brevicaulis *	8.0	Dehairing process	[5]
*Myrothecium verrucaria *	8.3	Feather degradation	[50]
*Chrysosporium keratinophilum *	9.0	Stable keratinase	[36]
*Trichoderma atroviride *	8.0–9.0	Feather degradation	[51]

Bacteria			
*Clostridium sporogenes *	8.0	Novel keratinolytic activity	[52]
*Microbacterium arborescens *	7.0	Feather degradation	[53]
*Fervidobacterium islandicum *	9.0	Feather degradation	[35]
*Kytococcus sedentarius *	7.0–7.5	Feather degradation	[54]
*Stenotrophomonas maltophilia *	7.8	Purification and characterization	[55]
*Kocuria rosea *	7.5	Feather degradation	[56]
*Xanthomonas maltophilia *	8.0	Purification and characterization	[57]
*Streptomyces thermoviolaceus *	8.0	Feather degradation	[58]
*Bacillus pumilus *	10.0	Purification and characterization	[59]
*Thermoanaerobacter keratinophilum *	8.0	Isolation of keratinophilic species	[34]
